# Subunit vaccines based on intimin and Efa-1 polypeptides induce humoral immunity in cattle but do not protect against intestinal colonisation by enterohaemorrhagic *Escherichia coli* O157:H7 or O26:H-

**DOI:** 10.1016/j.vetimm.2006.12.009

**Published:** 2007-03-15

**Authors:** P.M. van Diemen, F. Dziva, A. Abu-Median, T.S. Wallis, H. van den Bosch, G. Dougan, N. Chanter, G. Frankel, M.P. Stevens

**Affiliations:** a Institute for Animal Health, Compton, Berkshire RG20 7NN, UK; b Intervet UK Ltd., Walton Manor, Walton, Milton Keynes MK7 7AJ, UK; c The Wellcome Trust Sanger Institute, Hinxton, Cambridge CB10 1SA, UK; d Imperial College London, London SW7 2AZ, UK

**Keywords:** EHEC, enterohaemorrhagic *Escherichia coli*, EPEC, enteropathogenic *Escherichia coli*, Efa-1, EHEC factor for adherence 1, i.n., intranasal, CT-B, cholera toxin B subunit, Nal, nalidixic acid, Km, kanamycin, T-SMC, Sorbitol MacConkey agar supplemented with potassium tellurite, CFU, colony forming unit, Enterohaemorrhagic *Escherichia coli*, O157, O26, Cattle, Colonisation, Subunit vaccines, Immune response

## Abstract

Enterohaemorrhagic *Escherichia coli* (EHEC) infections in humans are an important public health concern and are commonly acquired via contact with ruminant faeces. Cattle are a key control point however cross-protective vaccines for the control of EHEC in the bovine reservoir do not yet exist. The EHEC serogroups that are predominantly associated with human infection in Europe and North America are O157 and O26. Intimin and EHEC factor for adherence (Efa-1) play important roles in intestinal colonisation of cattle by EHEC and are thus attractive candidates for the development of subunit vaccines. Immunisation of calves with the cell-binding domain of intimin subtypes β or γ via the intramuscular route induced antigen-specific serum IgG1 and, in some cases salivary IgA responses, but did not reduce the magnitude or duration of faecal excretion of EHEC O26:H- (Int_280_-β) or EHEC O157:H7 (Int_280_-γ) upon subsequent experimental challenge. Similarly, immunisation of calves via the intramuscular route with the truncated Efa-1 protein (Efa-1′) from EHEC O157:H7 or a mixture of the amino-terminal and central thirds of the full-length protein (Efa-1-N and M) did not protect against intestinal colonisation by EHEC O157:H7 (Efa-1′) or EHEC O26:H- (Efa-1-N and M) despite the induction of humoral immunity. A portion of the serum IgG1 elicited by the truncated recombinant antigens in calves was confirmed to recognise native protein exposed on the bacterial surface. Calves immunised with a mixture of Int_280_-γ and Efa-1′ or an EHEC O157:H7 bacterin via the intramuscular route then boosted via the intranasal route with the same antigens using cholera toxin B subunit as an adjuvant were also not protected against intestinal colonisation by EHEC O157:H7. These studies highlight the need for further studies to develop and test novel vaccines or treatments for control of this important foodborne pathogen.

## 1 Introduction

Enterohaemorrhagic *Escherichia coli* (EHEC) are zoonotic enteric pathogens of worldwide importance. Infections in humans may involve acute gastroenteritis and be complicated by haemorrhagic colitis and severe renal and neurological sequelae associated with the production of one or more Shiga toxins. Antibiotic use is contra-indicated in the treatment of such infections and current therapy is mostly supportive. Ruminants are an important reservoir of EHEC (Gansheroff and O’Brien, 2000), and human infections are frequently associated with direct contact with ruminants or their environment (Locking et al*.*, 2001; O’Brien et al., 2001). Consumption of meat, raw milk, vegetables, fruit or water contaminated with ruminant faeces is also a risk factor in sporadic cases of human EHEC infection (Caprioli and Tozzi, 1998). In Europe and North America EHEC infections are predominantly attributed to serotype O157:H7, but infections with non-O157 EHEC (especially of serogroups O26, O103, O111 and O118) are an emerging problem and indeed may be more common than those caused by O157 in some countries (Bettelheim, 2003). Stochastic simulation models predict that cattle are a key control point to reduce the incidence of EHEC infection in humans (Jordan et al., 1999), however until recently the host and bacterial factors influencing intestinal colonisation of cattle by EHEC were poorly understood.

EHEC strains produce intimin, an outer membrane adhesin encoded by the *eae* gene located in a chromosomal pathogenicity island termed the locus of enterocyte effacement (LEE; reviewed in Stevens and Wallis, 2005). Intimin mediates intimate bacterial attachment to enterocytes by binding to Tir, a bacterial protein which is translocated into host cells by a LEE-encoded type III secretion system. Intimin can also bind in vitro to β1-integrins and cell-surface localised nucleolin and these proteins can be detected proximal to adherent EHEC O157:H7 in vivo (Sinclair et al., 2006). Intimin is a key colonisation factor for EHEC O157:H7 in neonatal calves (Dean-Nystrom et al*.*, 1998), young and weaned calves (Dean-Nystrom et al., 1999; Vlisidou et al., 2006) and adult cattle and sheep (Cornick et al*.*, 2002). In addition, intimin influences the carriage and virulence of EHEC O157:H7 in streptomycin pre-treated mice (Judge et al., 2004), infant rabbits (Ritchie et al., 2003) and gnotobiotic and neonatal piglets (Donnenberg et al., 1993; Dean-Nystrom et al., 1998).

Studies with single and double *eae* and *tir* mutants of EHEC O157:H7 in calves and lambs have indicated that *tir* mutations are at least as attenuating as those affecting *eae*, suggesting that the intimin–Tir interaction, as opposed to binding of eukaryotic co-receptors, is of key importance (Vlisidou et al., 2006). Serological and phylogenetic analysis has identified at least six distinct intimin subtypes (designated Int-α, β, γ, δ, ɛ and θ) that vary in the sequence of the carboxy-terminal cell-binding domain (Adu-Bobie et al., 1998; Oswald et al., 2000; Zhang et al., 2002).

Although colonisation of calves by EHEC O157:H7 is intimin-dependent, EHEC O157:H7 (intimin subtype γ) has only been observed to form sparse microcolonies at distal sites in the intestines of calves (caecum, colon and rectum) with most bacteria being detected in the luminal contents (van Diemen et al., 2005). By comparison, in age-matched calves EHEC O26:H- (intimin subtype β) can be observed to adhere in extensive microcolonies at these sites, often covering entire villi, despite being shed in comparable numbers (van Diemen et al., 2005).

Intimin-specific antibodies can be detected in sera from patients convalescing from severe EHEC infection (Jenkins et al*.*, 2000; Li et al*.*, 2000; Karpman et al*.*, 2002). Antibodies directed against the cell-binding domain of intimin inhibit bacterial adherence to cultured epithelial cells (McKee and O’Brien, 1996; Gansheroff et al*.*, 1999; Carvalho et al., 2005) and porcine-intestinal explants (Girard et al*.*, 2006). Passively acquired intimin-specific antibodies also confer protection, since neonatal piglets allowed to suckle dams vaccinated intramuscularly with intimin-γ exhibit increased resistance to colonisation and intestinal damage following experimental inoculation with EHEC O157:H7 compared to piglets that suckled mock-vaccinated dams (Dean-Nystrom et al., 2002). Intimin-based subunit vaccines also confer protection upon the recipient; mice primed parenterally with the carboxyl-terminal portion of intimin-γ then orally fed transgenic intimin-γ-expressing plant cells, generate intimin-specific mucosal immune responses and shed EHEC O157:H7 for a shorter duration than mock-vaccinated animals (Judge et al., 2004). However, intimin-specific responses may be subtype-specific since immunisation of mice with the carboxyl-terminal domain of intimin-α from EPEC O127:H6 induced protection against a *Citrobacter rodentium* strain engineered to express intimin-α, but not to wild-type *C. rodentium* expressing intimin-β (Ghaem-Maghami et al., 2001). While it has been shown that intranasal immunisation of cattle with a carboxyl-terminal 64 kDa intimin polypeptide adjuvated with a low-toxicity derivative of *E. coli* heat-labile toxin induces antigen-specific serum IgG1 and salivary IgA (Yokomizo et al., 2002), the protective efficacy of intimin-based subunit vaccines in cattle has yet to be tested.

Another factor influencing colonisation of the bovine intestines is EHEC factor for adherence (Efa-1). Non-O157 EHEC, including serotype O26:H-, contain a full-length copy of *efa-1* while EHEC O157:H7 contains a truncated form which is predicted to encode the amino-terminal 433 amino acids of the protein (*efa-1*′) (Perna et al*.*, 2001). Mutation of *efa-1* in EHEC serotypes O5:H- and O111:H- significantly reduced faecal excretion and bacterial adherence to the colonic epithelium in experimentally infected calves (Stevens et al*.*, 2002b). Antibodies directed against the central and carboxyl-terminal portions of Efa-1 label the bacterial surface and inhibit adherence of rabbit enteropathogenic *E. coli* (EPEC) to cultured epithelial cells and this may indicate that Efa-1 is an adhesin per se (Badea et al*.*, 2003). However, *efa-1* mutations in EHEC O5:H- and O111:H- indirectly impair the expression and secretion of type III secreted proteins encoded by the LEE that are known to influence intestinal colonisation (Stevens et al., 2002b; Dziva et al., 2004; van Diemen et al., 2005). Mutation of the truncated *efa-1* gene of EHEC O157:H7 impaired adherence to cultured cells but did not significantly impair intestinal colonisation of calves (Stevens et al., 2004).

The aim of the present study was to assess the protective efficacy of subunit vaccines comprising of intimin and Efa-1 polypeptides against intestinal colonisation of cattle by EHEC strains of serotypes O157:H7 and O26:H- following parenteral and mucosal immunisation. The protection conferred by a formalin-inactivated EHEC O157:H7 bacterin was also assessed, since inactivated vaccines are effective in the control of other bacterial diseases including salmonellosis, pasteurellosis and coliform mastitis.

## 2 Materials and methods

### 2.1 Bacterial strains

EHEC O157:H7 strain EDL933 (*stx1*^+^, *stx2*^+^, *eae-*γ) was isolated in 1982 following an outbreak of haemorrhagic colitis in the USA (Riley et al*.*, 1983). EHEC O26:H- strain 193 (*stx1*^*+*^, *eae-*β) was isolated in 1962 in the USA from a calf with diarrhoea (Mainil et al*.*, 1987). Strains EDL933nal^R^ (Dziva et al., 2004) and 193nal^R^ (van Diemen et al., 2005) are spontaneous nalidixic acid resistant derivatives of EDL933 and 193, respectively, and exhibit normal growth and adhesion characteristics in vitro and efficiently colonise the intestines of calves. Strain STM2H2 is a kanamycin resistant derivative of 193nal^R^ that is fully colonisation proficient in calves (van Diemen et al., unpublished data). *E. coli* K-12 strain BL21 (DE3) Star cells were obtained from Novagen^®^ (Merck Biosciences Ltd., Nottingham, UK). Bacteria were routinely cultured using Luria-Bertani (LB) medium supplemented with the following antibiotics where appropriate: ampicillin (Amp) 100 μg/ml; nalidixic acid (Nal) 25 μg/ml; kanamycin (Km) 50 μg/ml. For oral inoculation studies, bacterial strains were amplified in brain heart infusion broth for 18 h at 37 °C with shaking.

### 2.2 Production and purification of recombinant proteins

The portion of the *eae* gene that encodes the carboxyl-terminal 280 amino acids of intimin was amplified by polymerase chain reaction from EHEC O26:H- strain 193 (Int_280_-β) and EHEC O157:H7 strain EDL933 (Int_280_-γ) using a conserved forward primer (Int-LIC-for: 5′-GAC GAC GAC AAG ATT ACT GAG ATT AAG GCT G-3′) and subtype-specific reverse primers (O26Int-LIC-rev: 5′-GAG GAG AAG CCC GGT TTA TTT TAC ACA AAC AG-3′ and O157Int-LIC-rev: 5′-GAG GAG AAG CCC GGT TTA TTC TAC ACA AAC CG-3′). The products were cloned in pET30-Ek/Lic (Novagen^®^) by a ligation-independent method as amino-terminal 6×His-S-tag fusions. The amino-terminal and central portions of Efa-1 from EHEC O111:H- strain E45035 (Efa-1-N, amino acids 1–993; Efa-1-M, amino acids 994–1896) and the truncated version of Efa-1 from *E. coli* O157:H7 (Efa-1′) were cloned in pET30-Ek/Lic as described elsewhere (Abu-Median et al., 2006).

Proteins were expressed in *E. coli* K-12 strain BL21 (DE3) Star cells which lack RNaseE to stabilise mRNA. The Overnight Express™ Autoinduction System I (Novagen^®^) was used to induce Int_280_-γ and Int_280_-β expression. Cell extracts were prepared using BugBuster^®^ (Novagen^®^) and the supernatant fraction mixed with His-Mag™ Agarose Beads (Novagen^®^) for affinity purification of the Int_280_ proteins as described by the manufacturer. Efa-1 proteins were expressed under IPTG induction in *E. coli* BL21 (DE3) Star and the proteins purified by affinity chromatography using HIS-Select™ Nickel Affinity Gel (Sigma, St. Louis, MO, USA) under denaturing conditions. Bacterial cell pellets were lysed by resuspension in 6 M guanidine hydrochloride and cell debris was removed by centrifugation. The supernatant was then equilibrated with sodium phosphate (0.1 M, pH 8.0) containing 8 M urea before applying to the affinity gel. Proteins were eluted with 0.1 M sodium phosphate, pH 4.5, 8 M urea, and dialysed against 200-fold volume pure water using Slide-A-Lyzer cassettes (Pierce Biotechnology, Inc., Rockford, IL, USA) for 2 h at 4 °C. The yield, size, stability and purity of the expressed proteins were assessed by 4–15% SDS-PAGE and Western blotting with Novagen^®^ monoclonal antibodies specific to the 6×His tag according to manufacturer's instructions. Protein concentrations were determined by the BCA method (Pierce Biotechnology, Inc.).

### 2.3 Vaccines and adjuvants

For intramuscular vaccinations (i.m.), Int_280_ and Efa-1 polypeptides were formulated in an aluminium hydroxide oil-based adjuvant (Alu-Oil; Intervet International BV, Boxmeer, The Netherlands) at 100 μg protein/dose. The mock (water) vaccine and an EHEC O157:H7 bacterin were also formulated in Alu-Oil. The bacterin consisted of formalin-inactivated EDL933nal^R^ grown statically for 6 h at 37 °C in a 5% CO_2_ atmosphere in Dulbecco's Modified Essential Medium supplemented with 10% (v/v) foetal calf serum and 25 mM HEPES (3.9 × 10^9^ cells/dose). These conditions are known to activate the expression of LEE-encoded proteins. The vaccines (2 ml) were delivered at a single site in the neck muscle.

For intranasal immunisations (i.n.), the relevant proteins (500 μg/dose) were mixed with cholera toxin B subunit (CT-B 100 μg/dose, Sigma) as a mucosal adjuvant. CT-B is known to potentiate immune responses against co-administered antigens in a variety of model systems (Rappuoli et al*.*, 1999; Douce et al*.*, 1997). The bacterin was mixed at 10^10^ cells/dose with 100 μg/dose CT-B. A syringe fitted with a device to create a fine aerosol was inserted as far as possible into the nostril and 2.5 ml of the solution was delivered into each nostril during inhalation.

### 2.4 Vaccination and challenge model

All animal experiments were performed in accordance with the Animals (Scientific Procedures) Act 1986 and were approved by the local Ethical Review Committee. Calves were housed in individual pens on straw and fed on milk replacer with free access to water, hay and weaner pellets. Two days before oral challenge the calves were transferred to security containment level 3 accommodation and housed in pens on shavings. Animals were monitored for EHEC prior to each immunisation and challenged by direct plating of faeces on Sorbitol MacConkey agar (Oxoid, Basingstoke, UK) containing 2.5 μg/ml potassium tellurite (T-SMC). It is acknowledged that T-SMC may not detect all EHEC, however, it is generally effective for the selection of the predominant serogroups O157 and O26. Presumptive EHEC colonies were screened for the presence of *stx1*, *stx2* and *eae* genes by colony PCR as described elsewhere (Stevens et al., 2004). Calves excreting EHEC or in generally poor health status were excluded from the study.

Established calf oral inoculation models using EDL933nal^R^ (Dziva et al., 2004) and 193nal^R^ (van Diemen et al., 2005) were used with minor modifications. In Trial 2, it was necessary to use a kanamycin-resistant derivative of strain 193nal^R^ (STM2H2) owing to a background of endogenous nalidixic acid and tellurite resistant (*stx1*, *stx2*, *eae* negative) bacteria in some of the calves. In total, 3 trials were performed with 12, 14-day-old conventional Friesian bull calves in each trial.

In Trial 1, on day 0 (14 ± 1 days old) and day 28 calves were vaccinated i.m. with Int_280_-γ (*n* = 4), Efa-1′ (*n* = 4) or mock (*n* = 4), followed on day 42 by oral challenge with 2.9 ± 0.78 × 10^10^ colony forming units (CFU) of EHEC O157:H7 strain EDL933 nal^R^.

In Trial 2, calves (14 ± 1 days old) were vaccinated i.m. with either Int_280_-β (*n* = 4), a mixture of Efa-1-N and M (*n* = 4; 100 μg/dose each), or mock (*n* = 4) on days 0 and 28, followed on day 42 by oral challenge with 2.8 ± 0.67 × 10^10^ CFU EHEC O26:H- strain STM2H2.

In Trial 3, calves (13 ± 1 days old) were vaccinated i.m. with a mixture of Efa-1′ and Int_280_-γ (*n* = 4; 100 μg/dose each), EHEC O157 bacterin (*n* = 4) or mock (*n* = 4), on days 0 and 28, combined with i.n. vaccination on days 28 and 42. The calves were challenged on day 56 with 1.93 ± 0.78 × 10^10^ CFU EDL933nal^R^.

After challenge, viable EHEC per gram of faeces were enumerated twice daily for at least 14 days post-inoculation by plating triplicate 10-fold serial dilutions of faeces onto T-SMC medium containing 25 μg/ml nalidixic acid (EDL933nal^R^-challenged animals) or T-SMC-Nal medium containing 50 μg/ml kanamycin (strain 193nal^R^-STM2H2 -challenged animals). The sensitivity of detection was 10^2^ CFU/g.

### 2.5 Immune parameters

Venous blood and saliva samples were collected before each vaccination and before challenge to monitor sero-conversion following vaccination. Enzyme-linked immunosorbent assays (ELISA) were developed to detect serum IgG1 and salivary IgA antibodies to intimin and Efa-1 polypeptides, CT-B and O157 lipopolysaccharide (LPS). For this purpose, 96-well microtiter plates (F96 Maxisorp, Nunc, Denmark) were coated overnight at 4 °C with 100 μl/well of 100 μg/ml of purified histidine-tagged recombinant protein (Int_280_-γ, Int_280_-β, Efa-1′, Efa-1-N, Efa-1-M), 1 μg/ml CT-B or 0.5 μg/ml LPS of EHEC O157:H7 in Carbonate buffer (pH 9.6). Pre-determined sample dilutions (serum 1:640, saliva 1:10) were added to the antigen-coated wells. After incubation for 1 h at room temperature and subsequent washing, wells were incubated with horseradish peroxidase-conjugated sheep anti-bovine IgG1 (1:5000, Serotec, Oxford, UK) for 1 h at room temperature. After further washing, tetramethylbenzidine substrate (TMB, Sigma, St. Louis, MO, USA) was added. Colour formation was stopped after 10 min with 2.5N sulphuric acid. The optical density was measured at 450 nm (OD_450_) with background correction of 620 nm.

In pilot experiments to assess cross-reactivity (e.g. responses of Int_280_-γ-vaccinated animals to Efa-1′) it became apparent that a component of the responses detected by ELISA was specific to the affinity tag or contaminants from the *E. coli* expression host. Thus the serum samples were pre-adsorbed against an irrelevant protein (the major fimbrial subunit of EHEC O157:H7 F9 fimbriae) expressed from pET30/EkLIC with the same affinity tag in the same *E. coli* host as described (Low et al*.*, 2006). This step abolished cross-reactivity and ensured that the responses measured were against the biologically relevant portion of the antigens used.

Western blotting assays using purified proteins were performed to confirm the specificity of the responses measured in the ELISA. In order to confirm that serum antibodies raised in calves vaccinated with the truncated recombinant proteins could bind to native protein, ELISAs were performed with sera of representative calves, essentially as above, using whole bacterial cells (wild type and Δ*eae* or Δ*efa-1* strains) and sera pre-adsorbed on Δ*eae*- or Δ*efa-1* mutants. Briefly, *E. coli* O157:H7 strain 85–170nal^R^ and its isogenic Δ*eae* mutant (Vlisidou et al., 2006), and rabbit EPEC strain 83/89 (known to express native Efa-1) and its isogenic *efa-1* mutant (Badea et al., 2003), were separately inoculated into DMEM supplemented with 25 mM HEPES buffer and incubated statically at 37 °C for 6 h. Bacteria were killed and fixed by addition of 0.5% (v/v) formalin, then harvested by centrifugation and standardized for the ELISA as described by Zhu et al. (2006). Sera were pre-adsorbed for 1.5 h at 37 °C on a rolling platform.

### 2.6 Statistical analysis

Data on faecal shedding and antibody response were statistically analysed for effect of vaccination by means of an *F*-test, with the data taken as repeated measurements (Proc Mixed, SAS, 1995). Bacterial recoveries were analysed after a ^10^log transformation. Differences were considered significant when *P* < 0.05. Subsequently, pairwise comparisons were performed at the overall 0.05 level of significance. Where relevant, relationships between the bacterial recoveries and immune parameters were orthogonally fitted by polynomial regression using Proc Mixed and Proc GLM (SAS, 1995).

## 3 Results

### 3.1 Trial 1: i.m. vaccination of calves with Int_280_-γ or Efa-1′ induces antigen-specific serum IgG but does not confer protection against intestinal colonisation by EHEC O157:H7

**Fig. 1:**
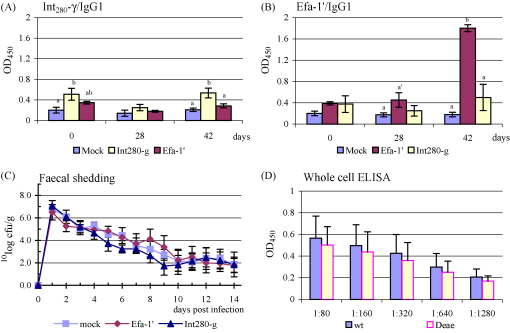
Serum IgG1 responses (mean ± S.E.M.) to Int_280_-γ (A) and Efa-1′ (B). Course of faecal excretion (mean ± S.E.M.) of EHEC O157:H7 strain EDL933nal^R^ following oral challenge of calves immunised i.m. with Int_280_-γ, Efa-1′ or mock-antigen (C). Serum antibodies were confirmed to bind native intimin-γ by ELISA using whole cells (wild type and Δ*eae* strains) and serum preabsorbed on *E. coli* O157:H7 Δ*eae* (D). Different letters (a and b) indicate pairwise significant difference (*P* < 0.05) within a testdate. Same letters but with an accent (′) indicate pairwise tendency towards significant difference (*P* < 0.1) within a testdate.

All vaccinated calves mounted a significant (*P* < 0.05) antigen-specific serum IgG1 response to the protein with which they were vaccinated (Fig. 1A and B). Sero-conversion was not detected in mock-immunised animals.

Three calves (1 Efa-1′ and 2 Int_280_-γ vaccinated) had antibodies recognising Int_280_-γ at the beginning of the experiment. On day 28 these antibodies were no longer detectable suggesting that they were maternal in origin. The response of the Int_280_-γ-vaccinated calves significantly increased to day 42 while the response of the Efa-1′ vaccinated calf remained low, indicating that maternal antibody may have been present. One Int_280_-γ-vaccinated calf did not significantly respond and the reasons for this are unclear.

The specificity of the responses was confirmed by Western blotting. The serum samples recognised proteins of the expected size and the intensity of staining increased with boosting as expected given the magnitude of the response detected by ELISA (data not shown).

Antigen-specific salivary IgA was not observed in calves immunised with Int_280_-γ or Efa-1′ (data not shown).

Calves were challenged 2 weeks after the second vaccination (day 42) with 2.9 ± 0.78 × 10^10^ CFU per calf of EDL933nal^R^. No significant differences in the magnitude or duration of faecal excretion were observed between the groups (Fig. 1C). The low responder calf shed comparable numbers of bacteria to other Int_280_-γ-vaccinated animals and those given mock antigen. Serum IgG1 from representative Int_280_-γ-vaccinated calves was preadsorbed on an *E. coli* O157:H7 Δ*eae* mutant and its reactivity against whole cells of wild type and Δ*eae* mutant *E. coli* O157:H7 measured by ELISA. Consistently lower reactivity was observed against cells lacking intimin, indicating that a portion of the serum IgG1 elicited by the truncated recombinant antigen recognises native intimin in the surface of *E. coli* O157:H7 (Fig. 1D).

### 3.2 Trial 2: i.m. vaccination of calves with Int_280_-β or a mixture of Efa-1-N and M induces antigen-specific serum IgG1 but does not confer protection against intestinal colonisation by EHEC O26:H-

**Fig. 2:**
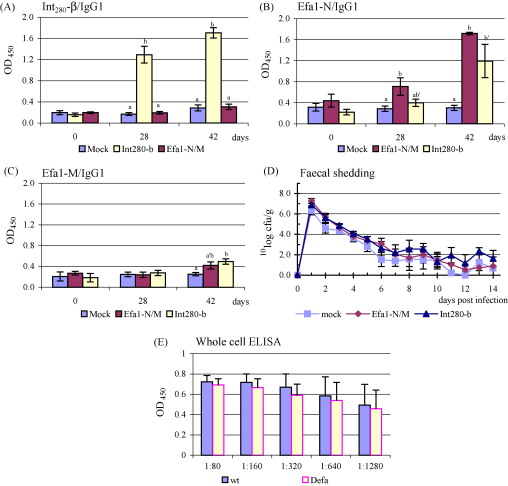
Serum IgG1 responses (mean ± S.E.M.) to Int_280_-β (A), Efa1-N (B) and Efa1-M (C). Course of faecal excretion (mean ± S.E.M.) of EHEC O26:H- strain STM2H2 following oral challenge of calves immunised i.m. with Int_280_-β, a mixture of Efa1-N and M, or mock-antigen (D). Serum antibodies were confirmed to bind native Efa-1 by ELISA using whole cells (wild type and *efa-1* strains) and sera preadsorbed on a rabbit EPEC *efa-1* mutant (E). Different letters (a and b) indicate pairwise significant difference (*P* < 0.05) within a testdate. Same letters but with an accent (′) indicate pairwise tendency towards significant difference (*P* < 0.1) within a testdate.

All vaccinated calves mounted a serum IgG1 response to the antigens used (Fig. 2A–C). The response against Int_280_-β and Efa-1-N was significantly (*P* < 0.05) higher than that of the mock vaccinated calves from day 28 onwards. The response to Efa-1-M was raised in the Efa-1 vaccinated calves on day 42 (*P* < 0.06) as compared with the mock calves. Pre-adsorption of the sera using his-tagged F9 protein expressed in *E. coli* as described in Section 2.5 significantly lowered, but did not completely abrogate the response of Int_280_-β vaccinated calves to Efa-1-N and M in this experiment.

Int_280_-β induced salivary IgA responses were significantly (*P* < 0.001) higher than those raised by mock-vaccinated animals on day 42. The salivary IgA responses to the Efa-1 polypeptides in animals immunised with Efa-1-N and M were not significantly different to controls (data not shown).

All calves were challenged 2 weeks after the second vaccination (day 42) with 2.8 ± 0.67 × 10^10^ CFU per calf of strain STM2H2. No significant differences in the magnitude or duration of faecal excretion were observed between the groups (Fig. 2D).

Serum IgG1 from representative calves vaccinated with Efa-1-N and M was preadsorbed on a rabbit EPEC *efa-1* mutant and its reactivity against whole cells of wild type and *efa-1* mutant bacteria measured by ELISA. Lower reactivity was observed against cells lacking Efa-1 (Fig. 2E), suggesting the presence of antibodies able to recognise native protein.

### 3.3 i.m. Priming of calves with a mixture of Int_280_-γ and Efa-1′ or an EDL933nal^R^ bacterin followed by intranasal boosting does not confer protection against intestinal colonisation by EHEC O157:H7

As administration of an intimin polypeptide via the intranasal route induces high titre serum IgG and salivary IgA responses in cattle (Yokomizo et al., 2002), we sought to determine if boosting parenterally primed animals via the intranasal route would improve immune responses and confer protection.

**Fig. 3:**
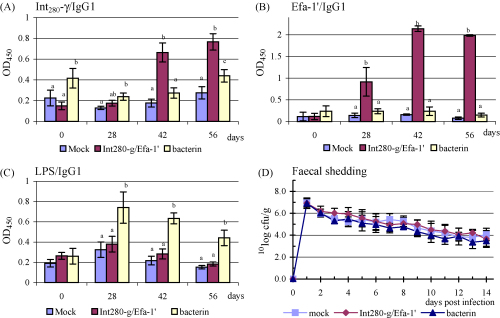
Serum IgG1 responses (mean ± S.E.M.) to Int_280_-γ (A), Efa-1′ (B) or EHEC O157 LPS (C). Course of faecal excretion (mean ± S.E.M.) of EHEC O157:H7 strain EDL933nal^R^ following oral challenge of calves primed i.m. with a mixture of Int_280_-γ and Efa-1′, formalin-inactivated EDL933 nal^R^ or mock-antigen, then boosted with the same antigens intranasally (D). Different letters (a and b) indicate pairwise significant difference (*P* < 0.05) within a testdate.

The Int_280_-γ/Efa-1′ vaccinated calves mounted significantly higher (*P* < 0.001) serum IgG1 responses against Int_280_-γ and Efa-1′ than the mock (Fig. 3A and B). The combined i.n./i.m. immunisation appeared to boost the anti-intimin IgG1 as compared with the results of Trial 1 (Fig. 1A). No further increase in IgG1 titre was observed following the final intranasal only immunisation.

The EDL933nal^R^ bacterin elicited significantly elevated IgG1 responses against Int_280_-γ on day 56 (*P* < 0.05; Fig. 3A) and against lipopolysaccharide (LPS) from EHEC O157:H7 from day 28 onwards (*P* < 0.001; Fig. 3C). The bacterin did not induce an IgG1 response to Efa-1′ (Fig. 3B).

Salivary IgA responses against Int_280_-γ and Efa-1′ were significantly higher in animals given the recombinant proteins than in mock-vaccinated animals on day 42. However, on day 56 the response of the controls were similar to those of the vaccinated calves (data not shown). No difference in salivary IgA were observed against LPS (data not shown).

**Fig. 4:**
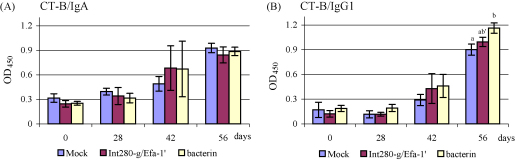
Salivary IgA (A) and serum IgG1 (B) responses (mean ± S.E.M.) against the intranasal adjuvant CT-B. Different letters (a and b) indicate pairwise significant difference (*P* < 0.05) within a testdate. Same letters but with an accent (′) indicate pairwise tendency towards significant difference (*P* < 0.1) within a testdate.

The intranasal adjuvant CT-B increased the salivary IgA response as compared with the before-intranasal-vaccination samples in all calves including the mock calves (Fig. 4A). Also a pronounced serum IgG1 response was detected (Fig. 4B) indicating that the vaccines were effectively delivered and recognised by the intranasal route.

Calves were challenged two weeks after the final immunisation with 1.93 ± 0.78 × 10^10^ CFU per calf of EDL933nal^R^. No significant differences in the magnitude or duration of faecal excretion between the groups were observed (Fig. 3D).

## 4 Discussion

Vaccination is one of several options for the control of enterohaemorrhagic *E. coli* in ruminants (reviewed in Stevens et al., 2002a). Few trials using rationally designed EHEC vaccines have been undertaken and to our knowledge the efficacy of whole-cell inactivated vaccines has not been assessed. Targeted and signature-tagged mutagenesis of bacterial genes has offered valuable insights into the molecular mechanisms influencing intestinal colonisation of cattle by EHEC and it is clear that proteins encoded by the LEE play key roles in persistence of both EHEC O157:H7 and O26:H-. Both the LEE-encoded adhesin intimin and the LEE-encoded type III secretion apparatus are known to be important in calves (Dean-Nystrom et al., 1998, 1999; Cornick et al., 2002; Dziva et al., 2004; van Diemen et al., 2005; Vlisidou et al., 2006). A vaccine based on the secreted fraction of EHEC O157:H7 induced humoral responses against key type III secreted proteins, as well as potent anti-lipopolysaccharide responses, and reduced the numbers of EHEC O157:H7 shed in the faeces, the number of animals shedding and the duration of shedding following experimental inoculation of calves (Potter et al., 2004). Vaccination also significantly (*P* = 0.04) reduced the prevalence of EHEC O157:H7 in a small scale trial using naturally exposed feedlot cattle (Potter et al., 2004). A vaccine derived from a *tir* deletion mutant was described as less efficacious than a preparation from the wild-type vaccine suggesting that disrupting intimin–Tir interactions may be fruitful (Potter et al., 2004). However, in subsequent trials in 218 pens of feedlot cattle in 9 feedlots in Alberta and Saskatchewan there was no significant association (*P* = 0.20) between vaccination with EHEC O157:H7 secreted proteins and pen prevalence of faecal EHEC O157:H7 following vaccination or prior to slaughter (van Donkersgoed et al*.*, 2005). These studies have re-invigorated efforts to develop effective and cross-protective vaccines for the control of EHEC.

The potential of intimin-based subunit vaccines was suggested following observations that antibodies against the carboxy-terminal domain inhibit bacterial adherence (McKee and O’Brien, 1996; Gansheroff et al*.*, 1999; Carvalho et al., 2005; Girard et al*.*, 2006). Subsequent studies in murine and porcine models have indicated that such vaccines reduce intestinal colonisation by EHEC to some extent, albeit that protection may only be subtype-specific if the variable cell-binding domain is used (Ghaem-Maghami et al., 2001; Dean-Nystrom et al*.*, 2002; Judge et al*.*, 2004). Immunisation of mice with the conserved domain of intimin (Int_388–667_) proved to be ineffective in a rodent model (Ghaem-Maghami et al., 2001), therefore intimin-based vaccines may need to be multivalent if they are to confer broad protection. Here, we have observed that while polypeptides based on the carboxyl-terminal domains of intimin-β and γ can induce serum IgG1 responses and variable salivary IgA responses when administered via the parenteral and mucosal routes, the responses do not confer protection against EHEC strains expressing homologous intimin types. The ability to induce humoral responses against intimin in cattle is consistent with the observations of Yokomizo et al. (2002), however to our knowledge we are the first to report the lack of efficacy of such responses in cattle.

While we were able to detect humoral responses against Efa-1 polypeptides, the biological activity of such antibodies is less well understood. Mutation of *efa-1*′ in EHEC O157:H7 was recently found to reduce bacterial adherence to cultured cells but had no significant effect on intestinal colonisation of calves and lambs (Stevens et al., 2004). This may partially explain the paucity of protection against EHEC O157:H7 infection that was conferred by this factor. While mutation of full-length Efa-1 in non-O157 EHEC does markedly impair persistence in calves (Stevens et al., 2002b), debate continues as to whether Efa-1 acts an adhesin per se in such strains or whether the attenuation reflects pleiotropic effects on the expression and secretion of LEE-encoded proteins.

Boosting of parenterally primed animals via the intranasal route further increased serum IgG1 responses to Int_280_-γ. Disappointingly, while salivary IgA responses against Int_280_-γ and Efa-1′ were significantly higher than controls at day 42 (after combined intranasal and intramuscular dosing on day 28), the responses on day 56 (after intranasal only immunisation on day 42) were not significantly greater than controls due to an increased response by the mock animals. The increase in salivary IgA titres over time was thought to be due to the relatively low specificity of IgA, which could be induced by a variety of other antigens encountered during the period. The extent to which mucosal immunisation was successful is therefore difficult to assess. The adjuvant cholera toxin B subunit (CT-B) induced potent serum IgG1 and salivary IgA responses in all (mock) vaccinated animals and was exclusively delivered via the intranasal route, indicating that opportunity existed for the co-administered antigens to have been recognised.

An inactivated vaccine comprising of formalin-killed EHEC O157:H7 strain EDL933nal^R^ proved to be ineffective when administered by the combined parenteral and mucosal regime, despite efficient induction of anti-LPS and anti-Int_280_-γ IgG1 responses. The bacteria were cultured under conditions known to activate expression of LEE-encoded proteins and incubated under static conditions to preserve fimbriae, EspA filaments and other surface-anchored molecules. At this stage we cannot preclude the possibility that such preparations may be effective when prepared under other conditions, when delivered by different routes or when tested against different challenge doses and/or natural exposure.

It is possible that the paucity of protection observed in the present studies reflects the high challenge doses used. However, our oral inoculation models have reliably detected the attenuating effect of mutations affecting Efa-1 (Stevens et al., 2002b), the LEE-encoded type III secretion system (Dziva et al., 2004; van Diemen et al., 2005), F9 fimbriae (Dziva et al., 2004) and intimin–Tir interactions (Vlisidou et al., 2006), despite using comparable inoculum sizes. Vaccinated animals shed similar numbers of organisms and for the same duration, even during substantial periods of the experiment when the number of organisms being shed was relatively low. The model was selected since challenge doses of 10^7^ CFU of EDL933nal^R^ or 193nal^R^ administered to duplicate naïve calves of the age to be used after vaccination (ca. 10 weeks old), failed to establish persistent infections (data not shown).

It is possible that the truncated recombinant antigens did not confer protection because they failed to induce high titres of antibody able to bind native surface-exposed intimin or Efa-1 and neutralise its activity. ELISA measurements using sera from calves vaccinated with Int_280_-γ or a mixture of Efa-1-N and M confirmed that a portion of the serum IgG1 response was able to recognise native protein on bacterial surfaces (Figs. 1D and 2E). The specificity of the response induced induced by Int_280_-β was not tested as an EHEC O26:H- Δ*eae* mutant strain was not available.

It is well established that in some cases effective immunity may be generated after infection but not after vaccination with purified inert molecules. This implies that there are aspects of the infectious process itself that initiate and/or potentiate the immune response. However, prior infection of calves with EHEC does not appear to confer protection against re-infection (Johnson et al*.*, 1996; Wray et al*.*, 2000) and responses against LEE-encoded proteins are weak or absent in experimentally infected and naturally colonised animals (van Diemen et al., unpublished observations). However, oral dosing of mice with intimin expressed in transgenic plant cells markedly potentiates humoral responses and confers protection in mice previously vaccinated with intimin by the intraperitoneal route (Judge et al., 2004). Thus, it remains possible that the antigens used herein may be suitable for immunisation of cattle provided appropriate exposure to the intestinal immune system can be achieved.

## 5 Conclusion

A whole cell-inactivated vaccine and subunit vaccines based on intimin and Efa-1 polypeptides induced serum IgG and variable salivary IgA responses following parenteral immunisation of cattle. Such responses did not confer significant resistance to intestinal colonisation by EHEC strains expressing the homologous antigens, even after boosting of such animals by the mucosal route.
